# Gene expression down-regulation in CD90^+ ^prostate tumor-associated stromal cells involves potential organ-specific genes

**DOI:** 10.1186/1471-2407-9-317

**Published:** 2009-09-08

**Authors:** Laura E Pascal, Young Ah Goo, Ricardo ZN Vêncio, Laura S Page, Amber A Chambers, Emily S Liebeskind, Thomas K Takayama, Lawrence D True, Alvin Y Liu

**Affiliations:** 1 Department of Urology, University of Washington, Seattle WA 98195, USA; 2 Institute for Stem Cell and Regenerative Medicine, University of Washington, Seattle WA 98195, USA; 3 Institute for Systems Biology, Seattle WA 98103, USA; 4 Department of Medicinal Chemistry, University of Washington, Seattle WA 98195, USA; 5 Department of Genetics, University of Sao Paulo's Medical School at Ribeirão Preto, Brazil; 6 Department of Pathology, University of Washington, Seattle WA 98195, USA; 7 LEP: Department of Urology, University of Pittsburgh Medical Center, Pittsburgh, PA 15232, USA

## Abstract

**Background:**

The prostate stroma is a key mediator of epithelial differentiation and development, and potentially plays a role in the initiation and progression of prostate cancer. The tumor-associated stroma is marked by increased expression of CD90/THY1. Isolation and characterization of these stromal cells could provide valuable insight into the biology of the tumor microenvironment.

**Methods:**

Prostate CD90^+ ^stromal fibromuscular cells from tumor specimens were isolated by cell-sorting and analyzed by DNA microarray. Dataset analysis was used to compare gene expression between histologically normal and tumor-associated stromal cells. For comparison, stromal cells were also isolated and analyzed from the urinary bladder.

**Results:**

The tumor-associated stromal cells were found to have decreased expression of genes involved in smooth muscle differentiation, and those detected in prostate but not bladder. Other differential expression between the stromal cell types included that of the CXC-chemokine genes.

**Conclusion:**

CD90^+ ^prostate tumor-associated stromal cells differed from their normal counterpart in expression of multiple genes, some of which are potentially involved in organ development.

## Background

Prostate stromal mesenchyme fibromuscular cells provide a regulatory extracellular matrix and direct epithelial differentiation and development through growth factors and androgen stimulation [1]. The critical role stromal cells play in prostate development has been demonstrated by co-implantation in animals of stem cells and stromal cells to achieve functional glandular development [2-7]. Although prostate cancer is epithelial in origin, there is a growing body of evidence suggesting that the stromal microenvironment plays a significant role in the cancer process [8-12]. Prostate tumor-associated or 'reactive' stroma is characterized by a decrease in smooth muscle cell differentiation and an increase in myofibroblasts and fibroblasts, with characteristics of a wound repair stroma [13]. Defining the gene expression changes in the stroma of prostate cancer has been the focus of several recent studies and is an important step in defining the underlying mechanisms of stromal-epithelial interaction in cancer. Previous studies have characterized gene expression profiles of tumor-associated stromal cells isolated by laser-capture microdissection (LCM) [14,15] and cultures established from histologically confirmed cancer tissues [16]. These studies have identified genes that are potentially involved in processes such as proliferation and angiogenesis. Current thought is that the tumor-associated stroma always co-exists with cancer [17], and that it may contribute to the gain of metastatic potential by tumor cells and the progression towards androgen-independence [12].

In this study, we sought to identify genes specific to prostate stromal cells that might function in organ specific stromal induction of epithelial development, and to isolate viable stromal cell populations associated with cancer by magnetic cell sorting (MACS) for gene expression analysis and comparison between these cells and their normal counterpart [18-20]. CD90/THY1 is a cell surface molecule expressed in a wide variety of cells including stem and progenitor cells [21-27]. It is thought to be involved in cell recognition, adhesion, and lymphocyte activation [26]. Elevated expression of CD90 has been found in the stromal cells of primary prostate cancer [28]. Previously, CD90^hi ^cells isolated from primary stromal cell cultures of prostate cancer were shown to differentially express several genes associated with tumor-promotion [16]. Here, we used differential expression of CD90 to isolate viable CD90-expressing stromal cells directly from prostate cancer specimens for gene expression profiling and comparison to normal tissue stromal cells. We also used differential expression of CD13 in the bladder stroma to isolate viable CD13-expressing stromal cells from bladder cancer specimens for further comparison. To date there has not been an established marker comparable to CD90 in prostate stroma that differentiates bladder tumor-associated stroma from normal. A population of CD13^+ ^cells in the so-called superficial lamina propria was regarded here as the prostate-equivalent bladder stromal cells because of its proximity to the urothelium [20]. These profiles provide important cell-type specific gene expression data for future *in vitro *differentiation and development studies to compare cancer-associated and normal tissue stromal cells. We used cell sorting rather than LCM because cell sorting results in a viable population that could subsequently be grown in cell culture whereas LCM cannot. Identification and isolation of a viable, sufficiently pure, cancer-associated stromal cell population from tumor specimens will provide an essential research tool for the study of prostate carcinogenesis.

## Methods

### Tissue specimens

The methods of tissue collection, expression data generation and analysis used in this study have been published previously [19,29,30]. The tissue samples consisted of prostate tissue specimens obtained from 13 patients undergoing radical prostatectomy under approval by the University of Washington Institutional Review Board. The same approach was used for both cancer-free and cancer-enriched (where at least 85% of the cells in the corresponding frozen section were of cancer) samples. Upon receipt of a radical specimen, 3-mm thick transverse sections were made of the prostate after inking the exterior surface. Cancer-free samples, weighing between 2 and 10 g, were harvested primarily from the anterior aspect of the prostate (transition zone) as described [19,20,31]. Corresponding frozen sections were histologically assessed to confirm the specimens were free of cancer. Cancer-enriched samples, weighing at least 0.1 g, were dissected from the opposing aspect of the non-fixed section adjacent to the block of tissue that had been sectioned. To minimize possible RNA degradation, organs resected in the operating room were immediately submerged in ice-cold saline solution. The pathology characteristics of the tumors from which stromal cells were obtained were as follows - 08-021: Gleason 5+4, T3a, 4.5 cc tumor volume; 08-028: Gleason 3+4, T2c, 2.5 cc; 08-032: Gleason 4+4, T3b, 27 cc. Whole tissue transcriptomes were generated from the following tumor samples - 05A: Gleason 3+4, T3a, 6 cc; 05B: Gleason 3+4, T2c, 3.4 cc; 05C: Gleason 4+5, T3a, 3 cc; 05D: Gleason 4+5, T3aN1, >5 cc; 05E: Gleason 3+4, T2a, 2.5 cc; and their matched non-cancer samples.

To obtain bladder stromal cells for analysis, tissue specimens were obtained from cystoprostatectomy specimens. Transverse sections of intact urethra and both ureters were taken to assess the surgical margins. After palpating the bladder externally to locate any masses and inking grossly concerning lateral surgical margin(s), the bladder was opened, avoiding transecting the tumor. Regions of bladder mucosae and wall that appeared grossly normal both visually and by palpation were identified as "normal" urinary bladder. An approximately 2 × 2 cm portion of bladder wall with minimal perivesicle fat was excised and cut into several pieces. Two pieces were histologically characterized to verify that the tissue collected was free of either invasive or in situ carcinoma. The others were used for cell sorting. The pathology characteristics of the tumor from which CB stromal cells were obtained was as follows: specimen 07A contained was a high-grade urothelial carcinoma, stage pT2N0.

For cell sorting, the collected specimens were processed within hours. The tissue was rinsed with Hanks balanced salt solution (HBSS) and minced for enzymatic digestion overnight at room temperature with 0.2% collagenase type I (Invitrogen, Carlsbad, CA) in RPMI-1640 media supplemented with 5% FBS and 10^-8 ^M dihydrotestosterone or serum-free media on a magnetic stirrer. The resultant cell suspension was filtered with 70-μm Falcon cell strainer, diluted with an equal volume of HBSS, and aspirated with 18-gauge needle. The single cell preparation was partitioned into stromal and epithelial fractions on a discontinuous Percoll density gradient (Amersham Pharmacia, Piscataway, NJ) as described previously [32,33].

### Western blot analysis of digestion media

HBSS-diluted tissue digestion media was centrifuged and the supernatant was collected. Protein concentrations were measured using Bradford Assay (BioRad, Hercules, CA). Sample buffer and 0.1 M DTT were added to amount of media containing 60 μg protein. The samples were heated to 70° for 10 min, electrophoresed on 4-20% gradient SDS-polyacrylamide gel (BioRad), and electrotransferred to PVDF membrane (Hybond-P, Amersham). The membrane was immersed in 5% nonfat dry milk in PBS-Tween for 30 min, and probed with tissue inhibitor of metalloproteinase 1 (TIMP1) antibody (1:500; MAB3300, Millipore, Temecula, CA) or CD90 antibody (1:500; 5E10, BD-PharMingen, San Diego, CA) for 60 min, followed by horseradish peroxidase conjugated anti-mouse IgG. After washing, the membrane was incubated with Luminol (Santa Cruz Biotechnology, Santa Cruz, CA) and immunoreactive bands were visualized using Biomax MR light film (Kodak, Rochester, NY). Prostate specific antigen (PSA) antibody (1:1000; A67-B/E3, Santa Cruz) was used for loading control.

### MACS cell isolation

Cell types were sorted using monoclonal antibodies specific for tumor-associated prostate stromal cells (CD90), tumor-associated bladder stromal cells (CD13) and normal bladder stromal cells (CD13) with MACS in the same manner as was previously used to generate the normal prostate stromal cell (CD49a) transcriptome [19]. The gradient-purified stromal cell fraction was resuspended in 100 μl 0.1% bovine serum albumin (BSA)-HBSS, and CD90-Phycoerythrin (PE) mouse monoclonal antibody (1:20, 5E10, BD-PharMingen) or CD13-PE (1:20, WM15, BD-PharMingen) added for 15 min at room temperature in the dark. The reaction was stopped by 1 ml 0.1% BSA-HBSS and centrifugation. The labeled cells were resuspended and 15 μl paramagnetic microbead conjugated anti-PE antibody (Miltenyi Biotec, Auburn, CA) was added for 15 min. After incubation the positive and negative cells were separated by AutoMACS cell sorter (Miltenyi Biotec) using double positive sort program.

### FACS analysis of sorted cells

Aliquots of positive and negative cell fractions were analyzed by fluorescence activated cell sorting (FACS) (Becton Dickinson, Mountain View, CA) to monitor the sort efficiency; only >85% CD90^+ ^and CD13^+^fractions were used for microarray experiments. The purity level was chosen based on our own observations (unpublished data) and studies by Szaniszlo *et al*., that showed that the transcriptome of a 75% pure sorted cell population is largely identical to that of a 100% pure population [34]. The sorted stromal cells were pelleted by centrifugation and lysed immediately in RLT (Qiagen, Valencia, CA). Total RNA was extracted for gene expression analysis using RNeasy (Qiagen).

### Gene expression profiling on Affymetrix DNA microarrays

Quality and concentration of RNA were determined using Agilent 2100 Bioanalyzer and RNA Nano or Pico Labchip (Agilent Technologies, Santa Clara, CA). Only RNA samples that were of sufficient concentration and showed no degradation as evidenced by distinct ribosomal bands at 18S and 28S were used for microarray experiments using the Human Genome U133 Plus 2.0 GeneChips (Affymetrix, Santa Clara, CA). The U133 Plus 2.0 array contained probesets representing 54,675 genes, splice variants, and ESTs. The GeneChips were prepared, hybridized, and scanned according to the protocols provided by Affymetrix (P/N 702232 Rev. 2). Briefly, 200 ng of RNA was reverse transcribed with poly (dT) primer/T7 promoter, and the cDNA was made double-stranded. *In vitro *transcription was performed to produce unlabeled cRNA. Next, first-strand cDNA was produced with random primers, and the cDNA was made double-stranded. *In vitro *transcription was performed with biotinylated ribonucleotides, and the biotin-labeled cRNA was hybridized to the GeneChips. The chips were washed and stained with streptavidin-PE using Affymetrix FS-450 fluidics station. Data was collected with Affymetrix GeneChip Scanner 3000. A total of 15 array datasets were obtained from the following sample types obtained from 10 patients: two CD90^+ ^prostate tumor-associated stromal, two CD13^+ ^normal bladder stromal, one CD13^+ ^bladder tumor-associated stromal, and five whole tissue prostate cancer and five normal tissue from matched pairs. Additionally, 8 array datasets were obtained from the following sample types obtained from 7 patients: five CD49a^+ ^normal prostate stromal (published previously [19]), and three CD26^+ ^prostate cancer (with one sample analyzed twice, Pascal *et al*., submitted). All datasets have been deposited in GEO with the following accession number: GSE17906.

### Microarray data analysis

Differential gene expression was determined by HTself, a self-self based statistical method for individual microarrays [35]. All possible combinations of pair-wise comparisons among experiments were taken to create sets of ratios. The test used virtual self-self experiments to derive intensity-dependent cutoffs. Accordingly, a probeset was considered significantly differentially expressed if all its log-ratio combinations were outside the 99.9% credibility cutoff. The computational analysis results were verified by dataset query of known differentially expressed genes. Pathway analysis of selected genes was done with KEGG. Functional and ontology enrichment analysis was performed using the DAVID web-based tool [36].

### Gene expression validation

Reverse transcriptase polymerase chain reaction (RT-PCR) and quantitative real-time PCR (qPCR) were used to validate expression obtained by DNA arrays. For each cell/tissue sample, 1 μg RNA was reverse transcribed with Superscript II RT (Invitrogen) at 50° for 50 min followed by 10 min at 70°. Gene-specific primers for PCR (Additional file 1) were designed to produce amplicons of 100-650 bp in size. PCR was carried out at 95°/30 s, 55°/30 s, 72°/1 min for 35 cycles. PCR products were resolved on 2% agarose gels. Smooth muscle actin (ACTA2), ribosomal protein RPL0 and GAPDH served as internal references for the various PCR experiments. Results were derived from specimens different from those used for transcriptome analysis.

## Results

### CP stromal cell transcriptomes

**Figure 1:**
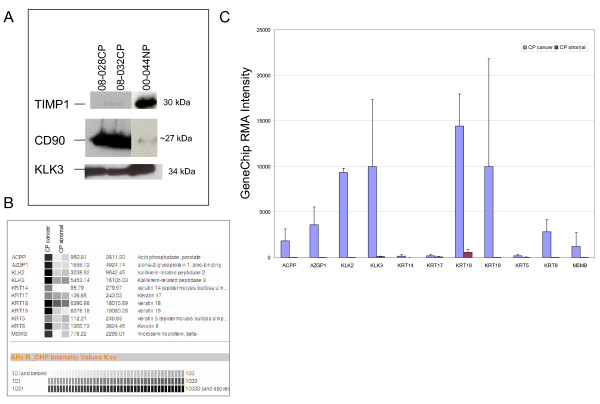
**Isolation of CD90^+ ^stromal cells from cancer tissue**. **A: **Tissue digestion media were probed for TIMP1 and CD90 proteins by Western blot analysis. Sample 00-044NP served as non-cancer control because 08-028NP and 08-032NP were not available. PSA was used as a sample loading control. **B: **The individual sorted CP stromal transcriptome datasets (second and third columns) contain minimal signals for the epithelial genes that are present in the cancer epithelial cell (CP cancer) transcriptome (first column) in virtual Northern blot format. Affymetrix signal values are represented on a gray scale. **C: **The expression levels of epithelial genes are below background (≤ 50 RMA) in sorted CP stromal cells.

The transcriptomes were determined for both CD90^+ ^prostate tumor-associated stromal cells and for CD13^+ ^tumor-associated and normal bladder stromal cells, the transcriptome for normal prostate stromal cells was determined previously [19]. For convenience, prostate tumor tissue was designated here as CP and non-cancer as NP; bladder tumor tissue was designated here as CB and non-cancer as NB. The transcriptome datasets of cells and tissue were deposited in our public database UESC [30]. For MACS, a minimum of 0.5 g tissue specimen was required. CD90^+ ^CP stromal cells were successfully sorted from 08-028 and 08-03; CD13^+ ^CB from 07A; and CD13^+ ^NB from 06A and 06B for Affymetrix GeneChip analysis. Sufficient RNA was not obtained from the CD90 sort of 08-021. CD90 was used based on prior finding that tumor-associated stromal cells were uniformly stained by the CD90 antibody within tumor foci [29]. In the bladder, CD immunohistochemistry showed that CD13 could define a population beneath the urothelium whereas the remainder was CD13-negative [20]. Prostate stromal cells are CD13^-^. CD13-PE was used to sort CD13^+ ^NB and CB bladder stromal cells for arrays. Western blot analysis of the collagenase digestion media for CD90 and TIMP1 was done to assess specimen purity. A 'pure' CP sample should have minimal reactivity to TIMP1, which is secreted by luminal cells and not by cancer cells [37]. Therefore, CP samples that showed minimal TIMP1 reactivity were relatively pure, i.e., with little 'contamination' of non-cancer elements as shown for 08-028CP and 08-032CP in Figure 1A. The increase in CD90 also confirmed that these CP specimens were from cancer. PSA was used as a sample loading control.

### Gene expression in CP vs. NP

**Figure 2:**
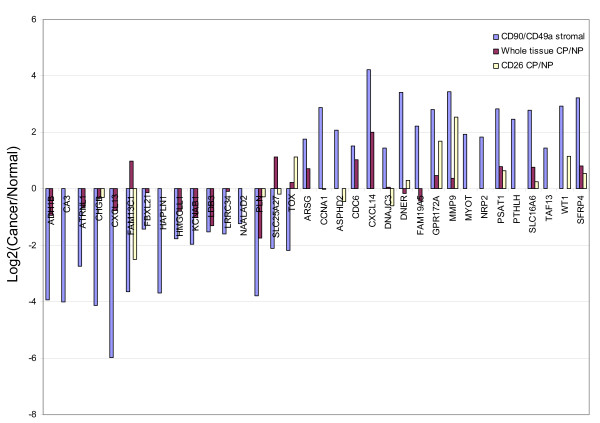
**Sorted cell and whole tissue transcriptome dataset comparison**. The most down-regulated (negative) and most up-regulated (positive) genes in sorted tumor-associated stromal cells are also similarly expressed, for the majority, in whole tissue comparison of CP to NP.

In order to identify genes unique to tumor-associated prostate stromal cells, differential gene expression was analyzed for CD90-expressing stromal cells from the two sorted cases and compared to previously determined CD49a^+ ^NP stromal cells, CD26^+ ^CP cells, and other prostate cell types determined previously in our lab [19,20,30]. The data were reported as robust multi-array average (RMA) [38] normalized Affymetrix signal intensities implemented in the in-house analysis pipeline SBEAMS [39], or as a composite value: X = log_2_(Cancer normalized intensity/Normal normalized intensity). These data were made available for download from the UESC database [18]. Dataset interrogation in UESC has been described previously [18,30]. Figure 1B and 1C showed that the CP stromal transcriptomes contained minimal signals for epithelial genes such as ACPP (prostatic acid phosphatase), AZGP1 (zinc α2-glycoprotein), KLK2/hK2, KLK3/PSA, MSMB/PSP_94_, and epithelial cell keratins (the cancer cell transcriptome [18], contained luminal KRT8, KRT18, KRT19 expression and not that of KRT5, KRT14, KRT17 for basal or intermediate cells [40]). Therefore, CD90 sorting was efficient enough to exclude cancer cells, the other major cell type of tumor tissue. Some of the most down-regulated and up-regulated genes in CD90^+ ^CP stromal cells compared to CD49a^+ ^NP stromal cells and verified by whole tissue and CD26^+ ^comparison are shown in Figure 2. Although the whole tissue transcriptome comparison of CP *vs*. NP showed decreased overall differential expression in these same genes, they were in agreement with the differential expression between sorted CP *vs*. NP. This decrease could in part be due to co-expression of these same genes by other cell types within the tissue, or to a 'tissue-masking' effect [20]. Furthermore, the expression levels of many of these tumor-associated stroma genes were below background (50 RMA) in both CD26^+ ^cancer and CD26^+ ^luminal epithelial cells.

Prostatic stromal cells are predominantly smooth muscle cells (SMC), characterized by desmin/DES, caldesmon/CALD1, α-smooth muscle actin/ACTA2 expression with few myofibroblasts (ACTA2, vimentin/VIM expression) and fibroblasts (VIM expression). In cancer, smooth muscle expression is decreased [41]. There is also reported loss of androgen receptor (AR) [42] and calponin (CNN1) [9] expression. Table 1 compares expression level of these genes and others as represented by gene array signal intensities and overall fold-change between NP and CP stromal cells and in agreement with whole tissue NP and CP transcriptome comparison. At least a 4-fold decrease for ACTA2, DES, CNN1, PENK, CNTN1, and 2-fold for CALD1 was found in CP stromal cells. Decrease in expression of these genes was also found in whole tissue comparison, to a lesser degree. No significant difference was seen for AR and VIM.

**Table 1:** Smooth muscle differentiation in CP stromal cells.

	CD49a+ NP	CD90+ CP	Fold change	Whole tissue NP	Whole tissue CP	Fold change
ACTA2	5399	1117	-4.83	5369	2882	-1.86
AR	211	182	-1.16	341	392	1.15
DES	144	27	-5.36	1573	482	-3.27
CALD1	1794	936	-1.92	1617	1103	-1.47
VIM	6519	6153	-1.06	1983	1700	-1.17
CNN1	2437	299	-8.16	5856	1928	-3.04
PENK	3896	998	-3.90	239	86	-2.79
CNTN1	791	197	-4.02	157	95	-1.66

Affymetrix signal levels are tabulated for the genes selected, and the fold decrease in expression in CP stromal cells compared to NP is indicated for sorted cells and whole tissue.

### Identification of potential organ-specific stromal genes in cancer

**Figure 3:**
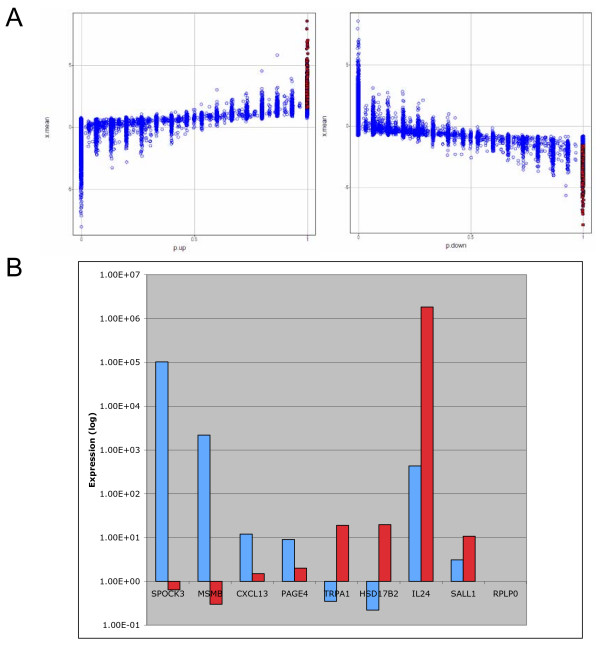
**Organ-specific stromal genes**. **A: **Up-regulated genes in the bladder (left) and the prostate (right) are identified by brown colored data points. The horizontal axis represent the fraction of spot replicates above (p.up) or below (p.down) the differentially expressed cutoff. The vertical axis represents the mean log-ratios. **B: **Shown are the qPCR results for prostate SPOCK3, MSMB, CXCL13, PAGE4, and bladder TRPA1, HSD17B2, IL24, SALL1. RPLP0 was used as reaction control. Light blue indicates prostate genes and red bladder genes.

Because of their central role in organ development, stromal cells might be expected to show organ-specific gene expression. Thus, by comparing the gene expression between prostate and, for example, bladder stromal cells, prostate-specific genes might be identified. A previous report described such a comparison using cultured prostate and bladder stromal cells, and cDNA microarray analysis [20]. In the current report, sorted prostate and bladder stromal cells were compared. Comparative dataset analysis between CD13^+ ^bladder stromal and CD49a^+ ^prostate stromal identified 91 bladder and 288 prostate differentially expressed genes (Figure 3). Among the genes identified, prostate expression of SPOCK3 (sparc/osteonectin proteoglycan/testican), MSMB, CXCL13 (chemokine ligand), PAGE4 (P antigen family member), and bladder expression of TRPA1 (transient receptor potential cation channel), HSD17B2 (hydroxysteroid 17-β dehydrogenase), IL24, SALL1 (*Drosophila *sal-like) were validated by qPCR (Figure 3). The prostate stromal dataset was further filtered by the previously determined prostate transcriptome datasets of CD104^+ ^basal, CD26^+ ^luminal, and CD31^+ ^endothelial. If a gene was present in 3 or more replicates, it was deemed as "present", and was removed from the CD49a stromal dataset. This resulted in a list of 50 stromal-specific genes that could be slotted into 39 functional categories using the software tools. Database query showed that expression of these 50 genes was enriched in sorted stromal cells as well as those obtained by laser-capture microdissection. The prostate stromal genes included CNTN1 (contactin), SPOCK3 and MAOB (monoamine oxidase), which all have been reported in the literature to have a function in development.

### Down-regulation of potential organ-specific stromal genes in cancer

**Figure 4:**
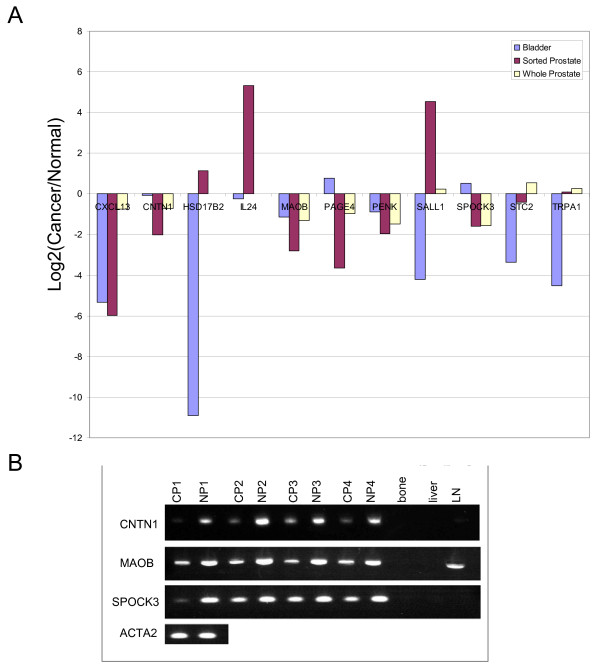
**Differential expression of organ-specific stromal genes in cancer**. **A: **Genes found to be differentially expressed in sorted tumor-associated stromal cells of bladder and prostate and prostate tissue. **B: **CP and NP are matched cancer and non-cancer prostate specimens; bone, liver and LN (lymph node) are prostate cancer metastasis specimens. CNTN1 is detectable in all NP, but it is down-regulated in CP; not detected in metastasis. A similar pattern is shown by MAOB and SPOCK3, but the differential expression is not as pronounced. MAOB is expressed in LN. cDNA quantity of each sample was monitored by ACTA2 (shown for CP1/NP1).

**Figure 5:**
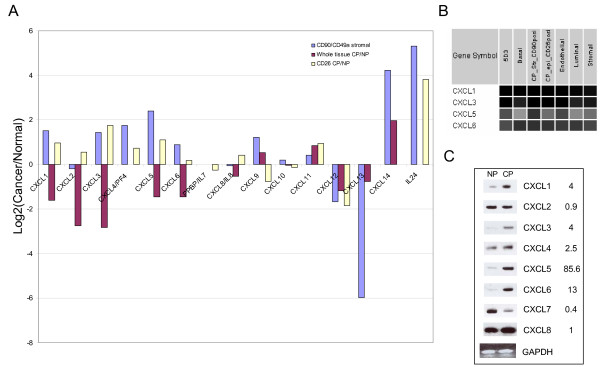
**Expression of CXC-chemokines in stromal cells**. **A: **Differential expression of CXC-chemokines in sorted CD90^+ ^CP *vs*. CD49a^+ ^NP stromal cells and comparison to whole tissue CP *vs*. NP and CD26^+ ^CP *vs*. NP. **B: **Virtual Northern display of expression levels of CXCL1, CXCL3, CXCL5 and CXCL6 in other prostate cell types (darker shading of the boxes indicates higher mRNA levels). **C: **RT-PCR verification of differentially expressed CXC-chemokines in sorted CD90^+ ^CP and CD49a^+ ^NP stromal cells.

Importance of the organ-specific stromal genes was suggested by their abnormal expression in cancer. Previously, we showed that expression of prostate-specific PENK was down-regulated in cancer [20]. Figure 4A shows the transcriptome data for the expression of identified prostate or bladder stromal genes in cancer *vs*. normal. Down-regulation was seen for CNTN1, CXCL13, MAOB, PAGE4, PENK, SPOCK3 in CP compared to NP as well as for HSD17B2, SALL1, TRPA1 for tumor-associated bladder stromal cells compared to normal bladder. The down-regulation of these putatively prostate stromal-specific genes was also evident in whole tissue CP *vs*. NP. With the exception of IL24 which is up-regulated 14-fold in CD26^+ ^CP *vs*. NP (see also Figure 5A), the expression level of these genes is either below background or very low in prostate epithelial cells (data not shown). RT-PCR analysis using matched CP and NP cDNA specimens showed that, like PENK, CNTN1 was detected in all NP samples, but its level was either decreased or undetectable in CP (and metastasis) samples (Figure 4B, bottom panel), while not as notably down-regulated for MAOB and SPOCK3. Smooth muscle actin (ACTA2) was used as control for stromal cell representation in these samples. Also notable was the increased CP expression of the bladder genes IL24 and SALL1 (IL24 was not down-regulated in CB). STC2 (stanniocalcin) is an example of a non-organ specific stromal gene that appeared to be down-regulated in CB (07-008); STC2 expression is higher in NB than NP [20].

### Expression of CXC-chemokines in tumor-associated stroma

Chemokines of the CXC family are involved in chemoattraction and activation of specific leukocytes in various immuno-inflammatory responses. They have also been shown to play key roles in neoplastic transformation and the passage of tumor cells through the endothelial vessel wall and extracellular matrix in several tumor types, including the prostate [43-47]. The expression of CXC chemokines was compared in sorted CP *vs*. NP stromal cells (Figure 5). Whole tissue comparison showed discrepancies for CXCL1, CXCL3, CXCL5 and CXCL6 (Figure 5A), these differences could in part be due to their high levels of expression in other prostate cell types (Figure 5B). The expression difference was assessed by RT-PCR of sorted prostate stromal cells (Figure 5C). The fold changes (in brackets) for CP/NP in array signal intensities for CXCL1 (4), CXCL2 (0.9), CXCL3 (4), CXCL4/PF4 (2.5), CXCL5 (85.6), CXCL6 (13), CXCL7/IL7 (0.4) and CXCL8 (1) were in good agreement with the RT-PCR results.

## Discussion

In prostate cancer, both the epithelial and stromal compartments can be distinguished by CD immunostaining from their normal/benign counterpart cell types [28]. CD immunostaining can also distinguish stromal cells of the prostate and the bladder [20]. Our data show that the stromal cell type in cancer differs from that in normal/benign in the expression of a significant number of genes, both down-regulated and up-regulated. A recently reported genome-wide analysis showed no somatic DNA changes in breast and ovarian tumor-associated stromal cells [48] so that DNA mutation could not likely contribute to the expression alteration. With expression alteration, the influence of CP stromal cells on epithelial differentiation and function is consequently different from that of NP stromal cells. The down-regulation of markers associated with SMC differentiation and of organ-specific genes suggests a loss of normal stromal signaling. We recently developed a co-culture system in which NP stromal cells were shown to induce differentiation of an embryonal carcinoma stem-cell line into a cell type with stromal cell gene expression, including the up-regulation of CNTN1, as well as that of epithelial markers [49]. This system could allow us to further explore the role of genes identified here in cell-cell interaction. Bladder stromal genes could be similarly tested. The involvement of stromal cells in cancer is a feature of the tissue organization theory of carcinogenesis [50], which equates cancer to inborn errors of development. Cancer is characterized by disruption of reciprocal intercellular signaling that maintains tissue organization in repair and turnover. For example, irradiation of breast stroma caused tumor formation from implanted non-irradiated mammary epithelial cells while non-irradiated stroma did not [51]. Of the down-regulated genes, PENK is a hormone known to function in development [52]. CNTN1 is known to mediate cell surface interactions in the development of the nervous system by signaling between axons and myelinating glial cells [53] and has been shown to promote cellular adhesion and invasion of lung cancer cells [54]. Also, CNTN1 has been shown to promote differentiation in CD90+ bone marrow stromal cells [55]. Both PENK and CNTN1 have been detected in the prostate in other studies [52,56], and cancer down-regulation of PENK was evident in published datasets [52]. SPOCK3 encodes a secreted protein involved in diverse steps of neurogenesis [57] and has been shown to inhibit tumor invasion [57]. MAOB catalyzes oxidative deamination of biogenic and xenobiotic amines with important roles in the metabolism of neuroactive and vasoactive amines in the central nervous system and peripheral tissues [58]. CNTN1 was detected here all NP samples analyzed, down-regulated in CP, and not detected in metastasis. A similar pattern is shown by MAOB and SPOCK3 to a lesser degree. MAOB was found to be expressed in lymph node metastases. The down-regulation of organ-specific genes appears to be a feature also of bladder cancer-associated stromal cells. Future analyses such as transcriptome determination of the various cell types in the bladder will be required to more fully understand the role of stromal-epithelial interaction in bladder carcinogenesis. TRPA1 functions in signal transduction and growth control, HSD17B2 may have a role in bone development, and SALL1 a role in kidney development. Interestingly, the bladder stromal genes SALL1 and IL24 appear to be expressed in CP stromal cells.

In addition, these cancer-associated stromal cells could also be the source of potential cancer biomarkers consequential to their production of secreted/extracellular protein products. For each cancer epithelial cell, there are about 5-10 associated CP stromal cells (True *et al*., submitted); as a result, a higher concentration of stromal-derived markers such as those identified here would be expected. The cell surface-anchored CD90 was detected in tissue digestion media [37] as well as in media of cultured stromal cells [59]. The increase in CD90 protein in cancer tissue was measured by quantitative proteomics [37] and was detectable by Western blot analysis. Therefore, secreted proteins like SFRP4 with a 12-fold increase in expression in tumor-associated stroma could potentially serve as markers for the presence of cancer. With appropriate antibodies, ELISA-type of assays could be developed for these marker proteins. Alternatively, multiplex-type of assays could be designed to detect these genes. The abnormal expression of Wnt pathway member SFRP4 and WT1 in stromal cells and that of other members in the epithelial cancer cells (Pascal *et al*., submitted) suggest a significant role of Wnt signaling in prostate carcinogenesis.

Two published datasets of tumor-associated *vs*. normal tissue stromal cells were available for comparison to ours. Genes scored as up-regulated (no down-regulated ones were found) by Richardson *et al*. [14] did not match those by Zhao *et al*. [16]. Expression of only a subset of these genes was scored as up-regulated in our datasets. The method of cell isolation could be a factor. Richardson *et al*. used laser-capture microdissection, but without immunostaining (e.g., with CD90) it is difficult to avoid capturing any NP stromal since the cancer-associated stroma does not extend beyond 10 cells from the tumor glands (True *et al*., submitted). Zhao *et al*. used cultured CP and NP stromal cells. Cell culturing is known to alter gene expression (e.g., expression of epithelial CD markers [31]). The datasets of Joesting *et al*. [60] were also generated from cultured cells but did not match well with those of Zhao *et al*. In addition, incorrect array probesets and non-agreement between array signals and protein expression determined by immunostaining could compromise data quality [18]. Our finding that genes involved in SMC differentiation and organ specificity were down-regulated in CP stromal cells is in line with the hypothesis that stromal cell differentiation in tumor is abnormal. The up-regulation of CD90 is notable because CD90 is a stem cell marker, and CD90^+ ^fibroblasts are considered to be a primitive cell type. Attenuated smooth muscle differentiation and down-regulation of developmental genes such as CNTN1, SPOCK3 and MAOB are an indication of such a possibility. Furthermore, the possibility exists that these tumor-associated stromal cells are similar to the normal stromal cells adjacent to the basal epithelium. Future comparison of the tumor-associated stromal cells to CD90^+ ^normal stromal cells may provide even greater insight into the role of stromal-epithelial interaction and how it might be disrupted in prostate carcinogenesis.

The cancer stroma contains not only fibromuscular cells but also CD45^+ ^white blood cells, CD31^+ ^endothelial cells of blood vessels, and nerve elements. These cell types could conceivably have a function in the cancer process as well [61,62]. Similar experimental analyses can be used to characterize and compare these cell types isolated from tumor and normal tissue.

## Conclusion

The results reported here provide evidence that gene expression of tumor-associated stromal cells differs from that of normal tissue stromal cells. Some of the genes affected have role in smooth muscle differentiation and reported function in organ development. These changes could in effect alter the functional property of stromal cells, in particular, in intercellular signaling in the tumor.

## Competing interests

The authors declare that they have no competing interests.

## Authors' contributions

LEP and YAG conceived and carried out experiments, analyzed data, and drafted the manuscript. RZNV analyzed microarray data and helped write the manuscript. TKT assisted with study design, and helped write the manuscript. LDT provided pathology analysis, assisted with study design and helped write the manuscript. LSP performed flow cytometry and cell culture. AAC performed Western blot experiments. AYL conceived experiments, study design and coordination, and revised the manuscript. All authors read and approved the final manuscript.

## Pre-publication history

The pre-publication history for this paper can be accessed here:

http://www.biomedcentral.com/1471-2407/9/317/prepub

## Supplementary Material

Additional file 1 **Primer sequences used for RT-PCR**. List of gene specific primers used for PCR. Click here for file
