# Supplementary material for: Gene expression down-regulation in CD90+ prostate tumor-associated stromal cells involves potential organ-specific genes
Source: BMC Cancer. 2009 Sep 8;9:317. doi: 10.1186/1471-2407-9-317 (PMC2745432; doi:10.1186/1471-2407-9-317)
Supplement: Additional file 1 — Primer sequences used for RT-PCR. List of gene specific primers used for PCR. [file 1471-2407-9-317-S1.doc]

**Supplemental File 1. Primer sequences used for RT-PCR.**

| **Gene** | **Sense Primer** | **Antisense Primer** |
| --- | --- | --- |
| SPOCK3 | AATGGCTCACCACAATCTCTCA | CCTGATCGAAGGGTTTTCCTG |
| MSMB | CTGGGCAGCGTTGTGATCTT | CCTGGAACTCCCTCATTAGGTAT |
| CXCL13 | CTCTGCTTCTCATGCTGCTG | TGAGGGTCCACACACACAAT |
| PAGE4 | GCTCCCGGTGAATCTCAGC | CGCTCACTCCGAGTCTTTTCC |
| TRPA1 | AAGGCCAAATTGAGCTAATGGA | TTCTACAGCACAATGCAGAGG |
| HSD17B2 | ACGGTATTTGCCGGAGTTTTG | TCGTGATGTCCATTTGGAGCA |
| IL24 | TTGCCTGGGTTTTACCCTGC | AAGGCTTCCCACAGTTTCTGG |
| SALL1 | AGGAAGCAAGCGAAGCCTC | CTTAGTAGGGCGACTCGGTTG |
| GAPDH | GCCTTCTCCATGGTGGTGAA | CACCATCTTCCAGGAGCGAG |
| RPLP0 | CAGATTGGCTACCCAACTGTT | GGGAAGGTGTAATCCGTCTCC |
| MAOB | GTTGAGCGTCTGATCCACCAT | TGTCATCCATTGTCCTCCAAAAG |
| ACTA2 | GTGTTGCCCCTGAAGAGCAT | GCTGGGACATTGAAAGTCTCA |
| CXCL1 | CTCTTCCGCTCCTCTCACAG | TCACGTTCACACTTTGGATG |
| CXCL2 | CTCAAGAATGGGCAGAAAGC | TCAAACACATTAGGCGCAAG |
| CXCL3 | GCAGGGAATTCACCTCAAGA | ACCCTGCAGGAAGTGTCAAT |
| CXCL4 | GCGCTGAAGCTGAAGAAGAT | GTCCGGCCTTGATCACCT |
| CXCL5 | ACCCAGGGAAGACAAGAAGG | CAGTGATTCCTGGCTCACAC |
| CXCL6 | GTCCTGTCTCTGCTGTGCTG | AACTTGCTTCCCGTTCTTCA |
| CXCL7 | TCCTCCACCAAAGGACAAAC | TTTCCTCCCATCCTTCAGTG |
| CXCL8 | CAGGAATTGAATGGGTTTGC | AACCAAGGCACAGTGGAAC |
